# The interaction of Ag_2_O nanoparticles with *Escherichia coli*: inhibition–sterilization process

**DOI:** 10.1038/s41598-021-81305-5

**Published:** 2021-01-18

**Authors:** Danqing Li, Shuai Chen, Ke Zhang, Nan Gao, Miao Zhang, Gadah Albasher, Jiangfan Shi, Chuanyi Wang

**Affiliations:** 1 School of Environmental Science and Engineering, Shaanxi University of Science and Technology, Xi’an, 710021 China; 2 Department of Materials Science and Engineering, Nano Institute of Utah, University of Utah, Salt Lake City, UT 84112 USA; 3 School of Pharmacy, Jiangxi Science and Technology Normal University, Nanchang, 330013 China; 4 Zoology Department, College of Science, King Saud University, Riyadh, 11451 Saudi Arabia

**Keywords:** Biochemistry, Bioinorganic chemistry

## Abstract

Silver-based antibacterial agents have obtained wide attention due to the fact that bacteria in the environment is ubiquitous, which has become one of the most difficult problems for human health. However, the antibacterial mechanism and process are still inconclusive. Here, Ag_2_O nanoparticles (NPs) with uniform spherical morphology and small size (around 30 nm) were prepared. The as-prepared Ag_2_O NPs induced high antibacterial activity (100% inhibition ratio) against *E. coli*. A two-step antibacterial process was proposed and confirmed, which divided into inhibition and sterilization steps. The optical density measurement, malondialdehyde concentration detection, morphologic imaging with electronic microscopy and Fourier transform infrared spectroscopic analysis unveiled the interaction of Ag_2_O NPs with *E. coli*, which verified the inhibition–sterilization process we proposed.

## Introduction

With the outbreak of infection caused by different harmful bacteria existing anywhere and the dramatically increasing antibiotic resistance, antibacterial treatments obtained much attention in recent years^1,2^. Therefore, various antimicrobial strategies have been developed, such as high-temperature, ultraviolet, photocatalytic^3,4^, chemical and other sterilizing techniques. Among them, chemical antibacterial techniques have attracted much interest due to their advantage in balancing low cost and high efficiency^5–8^. In recent years, as one of the chemical antibacterial techniques, antibacterial nanomaterials have become promising candidates for antibacterial application owing to their high specific surface area as well as unique chemical and physical properties^9,10^. Many nanomaterials such as ZnO, CuO, TiO_2_ and Ag_2_O showed great performance in antibacterial and were used as antibacterial agents^11–14^. Among them, Ag-based antimicrobial nanoparticles (NPs) have earned the most extensive applications due to their excellent antibacterial efficiency for a wide range of bacteria^15–17^. Thus, Ag-based NPs have been employed as an antiseptic component in many medical devices, food package and environmental purification process^18–20^.

It is difficult to balance the cost and sterilization efficiency for most antibacterial agents, which otherwise require supporting components or complex preparation process, for instance, using surfactants to increase solubility in organic phase^9^, inducing graphene oxide as supporting substrate for Ag NPs to improve dispersibility^21^ and involving polymers to enhance the flexibility and extensibility^22^. Additionally, achieving high antibacterial ratio also demands high dose of agents around μmol L^−1^^23^, and at least 2 h long sterilization time^24^. Furthermore, the antibacterial mechanism and process still remain in debate. Three types of mechanisms have been proposed in literature: (1) oxidative stress causing by reactive oxygen species (ROS) generated, (2) interaction of Ag^+^ with thiol groups in proteins, (3) the destruction of the bacteria cells via strong affinity interaction between Ag^+^ and cell membrane, little experimental evidence has been reported at the molecular level. More importantly, direct experimental confirmation on the process of bactericidal is still scarce^22,23,26^. Most studies only focused on the changes of bacteria cells caused by antimicrobial agents, while paying little attention to the influence of antibacterial materials themselves^25–27^.

In this work, an antibacterial agent based on the pure Ag_2_O NPs which was synthesized via a simple wet chemical method. Almost 100% inhibition ratio against *E. coli* was obtained, and the effect of preparation conditions on the properties of Ag_2_O NPs was explored. It was inferred that the antibacterial performance of Ag_2_O NPs critically depended on the interaction between Ag_2_O NPs and cell membrane, as well as the intrinsic chemical and physical properties of Ag_2_O NPs themselves. The high antimicrobial activity was evidenced by the optical density value at 600 nm. The inhibition–sterilization antimicrobial process was proposed and confirmed by spectral and microscopic analyses, including the bacteria growth curve monitored in 2 h, electronic microscopic images of morphologic change and Fourier transform infrared spectroscopic analysis of the cells. The increased concentration of malondialdehyde (MDA) also proved the lipid peroxidation of cell membrane occurred.

## Experimental section

### Materials

Silver nitrate (AgNO_3_, A.R.), potassium persulfate (K_2_S_2_O_8_, A.R.), potassium hydroxide (KOH, A.R.), podium phosphate monobasic dehydrate (NaH_2_PO_4_·2H_2_O, ≥ 99.0%), sodium phosphate dibasic dehydrate (Na_2_HPO_4_·7H_2_O, ≥ 98%), sodium chloride (NaCl, ≥ 99.5%) and sodium hydroxide (NaOH, ≥ 98.0%) were purchased from Aladdin. Tryptone (BR), agar powder (BR) and yeast extract powder (BR) were bought from AOBOX. Purified gram-negative *E. coli* and purified gram-positive *S.* *aureus* were offered by Environmental Engineering Microbiology Laboratory in School of Environmental Science and Technology, Shaanxi University of Science and Technology, China. The bacteria mentioned above were purified twice before experiments.

### Synthesis and characterization of Ag_2_O NPs

**Scheme 1:**
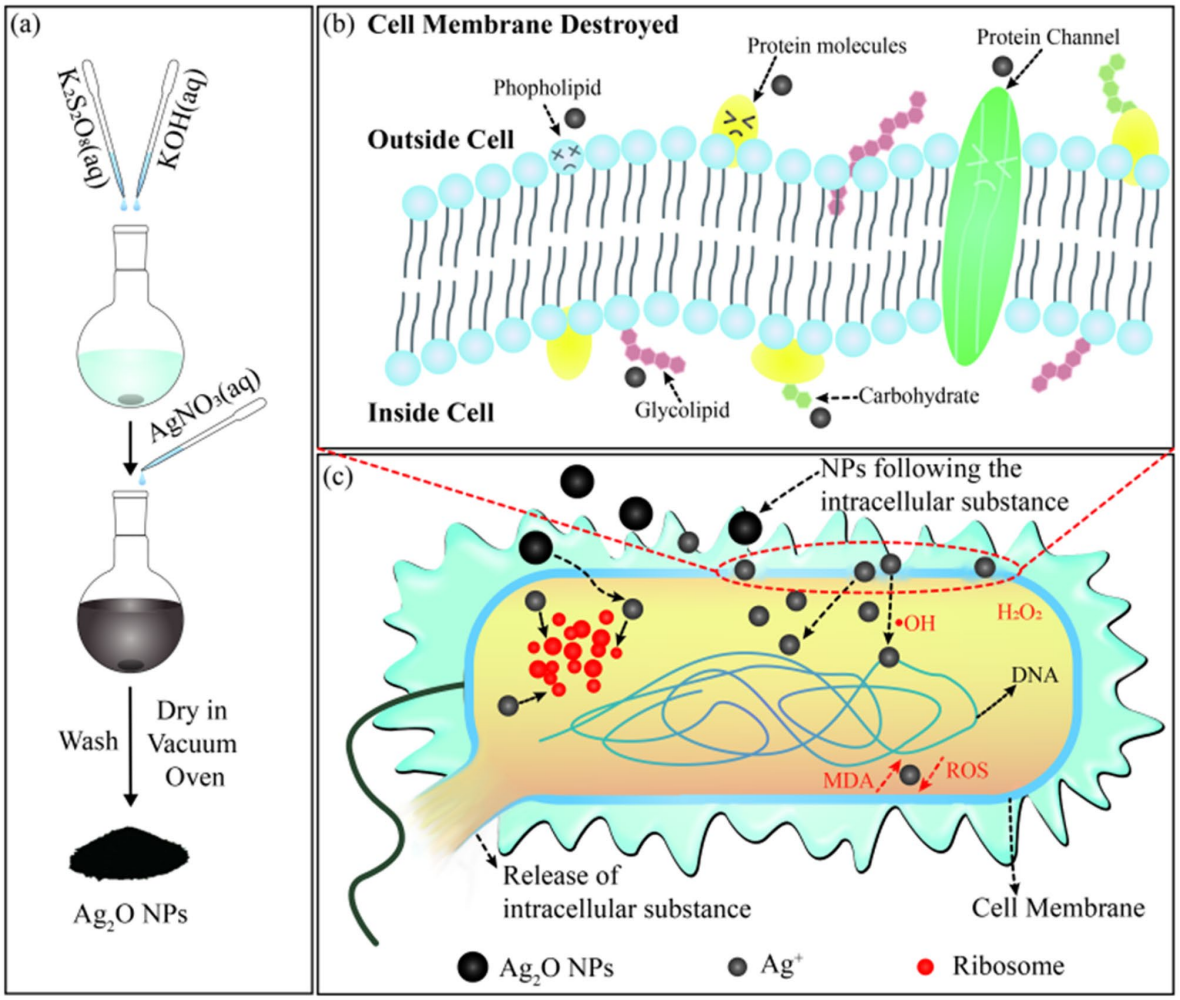
Schematic diagrams of (**a**) synthesis of Ag_2_O NPs, (**b**) Ag_2_O NPs induced cell membrane destruction, and (**c**) interaction between Ag_2_O NPs and *E. coli*.

The facile synthesis process of Ag_2_O NPs is schematically illustrated in Scheme 1a. 50 mL of 0.2 M AgNO_3_ (aq) was added with a mixture of 25 mL, 0.2 M K_2_S_2_O_8_ (aq) and 20 mL of 1 M KOH (aq) under magnetic stirring, and then kept the mixture solution stirring for different times at gradient temperatures. Black precipitation was formed immediately, then collected samples via centrifugation and washed them using deionized water for several times until the concentration of total ions in the supernatant was less than 10 ppm. Subsequently, the final black solid product was dried in vacuum drying oven for 10 h at 60 °C.

The powder X-ray diffraction (XRD, Panalytical) with Cu Kα radiation was used to obtain composition and phase information. Morphologies were characterized by scanning electron microscopy (SEM, FEI Verios 460). Thermostability was tested by thermogravimetric analyzer (TGA, Q500) under nitrogen atmosphere with heating rate of 10 °C/min. High resolution transmission electron microscopy (HRTEM, JEM 2100F) with an acceleration voltage of 200 kV was carried out to obtain the morphology and crystal facet information, and Fourier transform infrared spectrometer (FTIR, Bruker) was used to record the structural change before and after antibacterial treatment. Biological samples were freeze-dried for 20 h before test.

### Antibacterial activity test

Paper-disk diffusion method was used to determine the antibacterial activity of Ag_2_O NPs qualitatively, and to compare with two traditional antimicrobial agents. Gram-negative bacteria *E. coli* and gram-positive bacteria *S.* *aureus* were used as model bacteria for antibacterial test. After twice purification, the bacterial cells were cultured in a broth at a constant temperature of 37 °C to achieve to logarithmic growth phase, and the bacterial solution was dropped to the solid medium by a plate coating method. Filter papers (d = 1.5 cm) were immersed in a 0.01 wt% Ag_2_O NPs suspension, 0.01 vol% H_2_O_2_ solution, 0.01 vol% absolute ethanol for 2 min, respectively, taken out and then air-dried. Afterwards, filter papers were placed as an equilateral triangle on the same solid medium coated with a bacterial suspension. Subsequently, after incubated at 37 °C for 24 h, the appearance of inhibition zones was observed.

To quantitatively evaluate the antibacterial activity of Ag_2_O NPs, gram-negative bacteria *E. coli* was used as the model bacteria. The bacterial cells were cultured in broth at 37 °C for 6 h, then diluted with (phosphate buffer solution) PBS of pH 7.4 until optical density value at 600 nm (OD_600_) = 1.40–1.60, measured by Bio Tek (synergy/H1), and then Ag_2_O NPs were added. In sequence, culture medium was taken every 2 h to test OD_600_ in 96-well plate, and the growth curve was plotted within 12 h. The first value of OD_600_ is treated as zero to correct each curve that begins from the same point. The inhibition ratio can be calculated according to the equation as follows:

1 $$\mathrm{Inhibition\,ratio }\,\left(\mathrm{\%}\right)=\frac{{OD}_{control group}-{OD}_{test group}}{{OD}_{control group}}\times100\mathrm{\%}$$

The minimum inhibitory concentration (MIC) and the minimum antibacterial concentration (MBC) are the important indicators for measuring the antibacterial activity of a bactericidal agent. *E. coli* was cultured to a logarithmic growth phase firstly, and then Ag_2_O NPs was added in a gradient. (In this experiment, Ag_2_O NPs prepared at 60 °C/10 min were added by 5 μg mL^−1^, 10 μg mL^−1^, 20 μg mL^−1^, 30 μg mL^−1^, 40 μg mL^−1^ and 50 μg mL^−1^ respectively). After cultured at 37 °C for 24 h, each MIC was determined by visually locating the cells with no bacterial growth and the lowest concentration of the sample solution. By drawing the supernatant of the clarified samples in MIC experiment in solid medium, culturing at 37 °C for 24 h again, the minimum concentration at which no colony occurred or their number less than 10 was obtained as the MBC.

Before experiments, all required supplies (e.g. culture dishes, media, PBS solution, pipette tips, graduated cylinders, deionized water, etc.) were sterilized in autoclave. All above operations were carried out in clean bench, and all experiments were carried out with three parallel tests to eliminated possible error.

### Antibacterial process study

To in-depth investigate the bactericidal process of Ag_2_O NPs, four tests were taken: (1) environmental scanning electron microscope (E-SEM, FEI Q45) was applied to observe the morphological changes of bacteria; (2) malondialdehyde (MDA) will be generated inside the bacteria cells once membrane is destroyed. MDA kit (Nanjing Jiancheng Bioengineering Institute) was used to measure the content change; (3) XRD planes information and HRTEM lattice fringes information were used to represent how Ag_2_O NPs natural properties influenced antibacterial activity; and (4) FTIR was used to reflect the structural changes of *E. coli* cell and Ag_2_O NPs. After antimicrobial process, Ag_2_O NPs were collected from broth, washed three times by deionized water, and then dried in vacuum oven at 60 °C for 6 h. The cells as collected after sterilization were freeze–dried at least 20 h before FTIR test.

## Results and discussion

### Structure and morphology of Ag_2_O NPs

**Figure 1:**
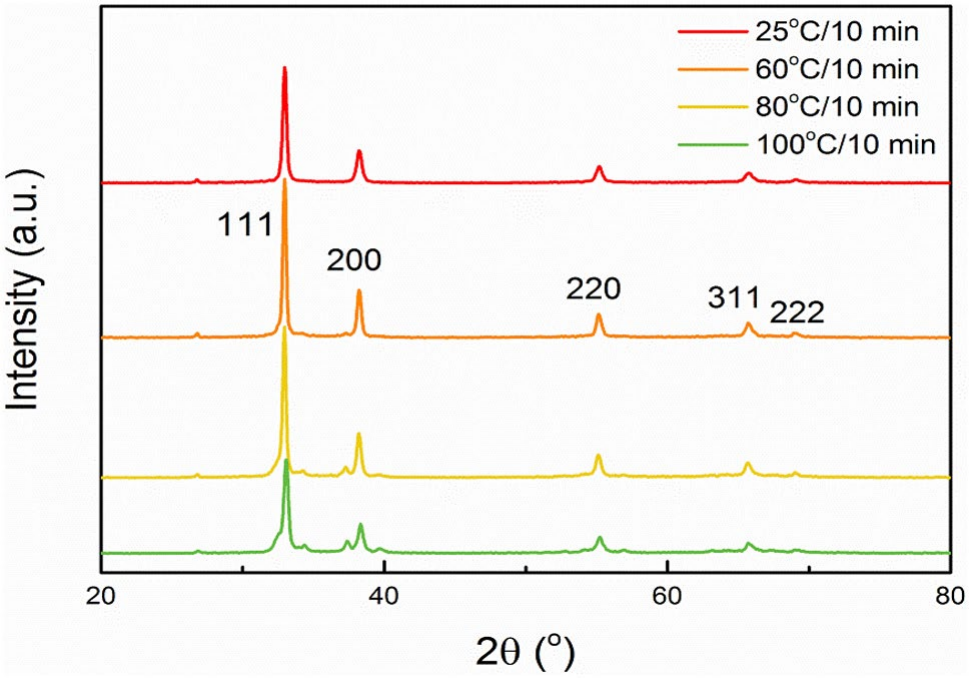
XRD patterns of Ag_2_O NPs synthesized under the reaction time of 10 min, and the temperatures are 25 °C, 60 °C, 80 °C, 100 °C, respectively.

**Figure 2:**
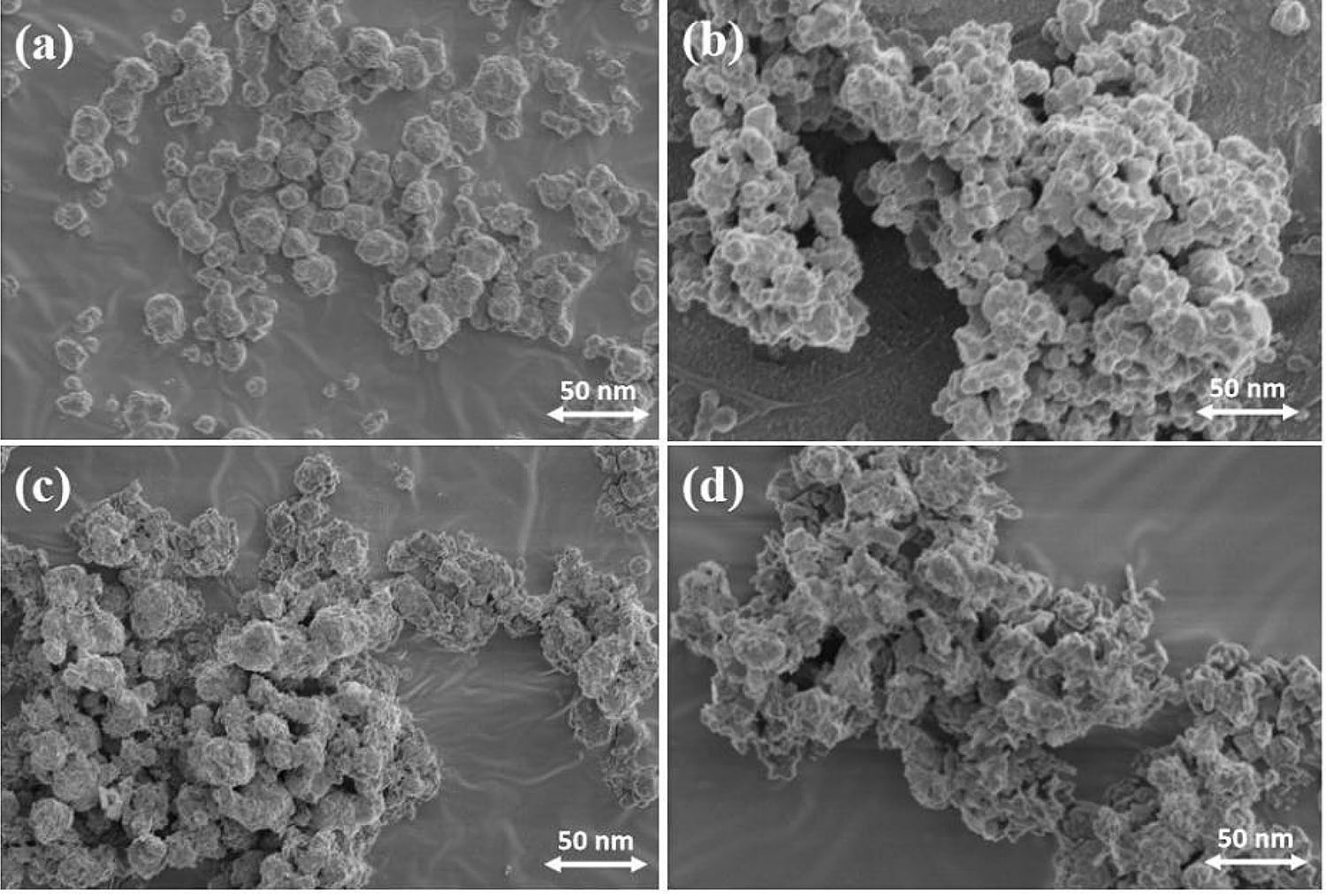
SEM images of Ag_2_O NPs prepared at different temperatures, (**a**) 25 °C, (**b**) 60 °C, (**c**) 80 °C, (**d**) 100 °C.

Synthesis temperature was adjusted carefully to control the crystal structure. The crystalline characters of Ag_2_O NPs were influenced by the reaction temperatures, which can be inspected from XRD patterns as shown in Fig. 1. Overall, five distinct diffraction peaks of Ag_2_O NPs appear at 2θ = 32.98°, 38.24°, 55.24°, 65.76° and 69.09° in four XRD patterns. The upper two XRD patterns show that Ag_2_O NPs have good crystallinity at 25 °C and 60 °C due to no obviously miscellaneous peaks appearing. Although the position of characteristic diffraction peaks are not obviously changing in 80 °C and 100 °C samples, slight miscellaneous peaks near the main diffraction peaks appear, especially around the (111), (200) and (220), which indicates undesirable microscopic defects therein. The grain sizes can be derived from XRD patterns, which are 29.7 nm (25 °C), 36.4 nm (60 °C), 37.2 nm (80 °C), 38.8 nm (100 °C), respectively. With the gradual increase of the temperature, the grain size increases slightly. However, the aggregation is more likely to occur due to too small size as shown in Fig. 2, which may have negative impact on the activity. Moreover, when the Ag_2_O NPs preparation temperature was increased to 60 °C, higher intense (200) diffraction of Ag_2_O NPs was observed than that of the sample synthesized at 25 °C. The (200) diffraction peak should be ascribed to high orientation of {100} crystal facet of Ag_2_O NPs^28^. From the previous work^28^, it has been proved the {100} crystal facet of Ag_2_O NPs is more active, which may makes better performance.

Preparation temperature also influences the morphology of Ag_2_O NPs as reflected by the SEM images shown in Fig. 2. With the synthesis times and temperatures changing, the morphology and dispersibility of the Ag_2_O NPs were changed gradually. It can be seen from the Fig. 2a, Ag_2_O NPs (25 °C/10 min) owns the best dispersibility and clear spherical shape. In contrast, although Ag_2_O NPs (60 °C/10 min) also have uniform spherical shape, most of them flock together (Fig. 2b), which may arise from their much smaller particle sizes and higher surface activity. When the temperatures continue rising, Ag_2_O NPs (80 °C/10 min) show much rough crystal surfaces with non-uniform sizes (Fig. 2c), while much severely for Ag_2_O NPs (100 °C/10 min) (Fig. 2d). From the result of the size distribution of Ag_2_O NPs (Fig. S1), it can be known the average size of each sample, which are 29.80 nm (25 °C/10 min), 37.60 nm (60 °C/10 min), 38.03 nm (80 °C/10 min), 39.28 nm (100 °C/10 min), respectively. The results above are nearly consistent with the results from XRD patterns in Fig. 1.

**Figure 3:**
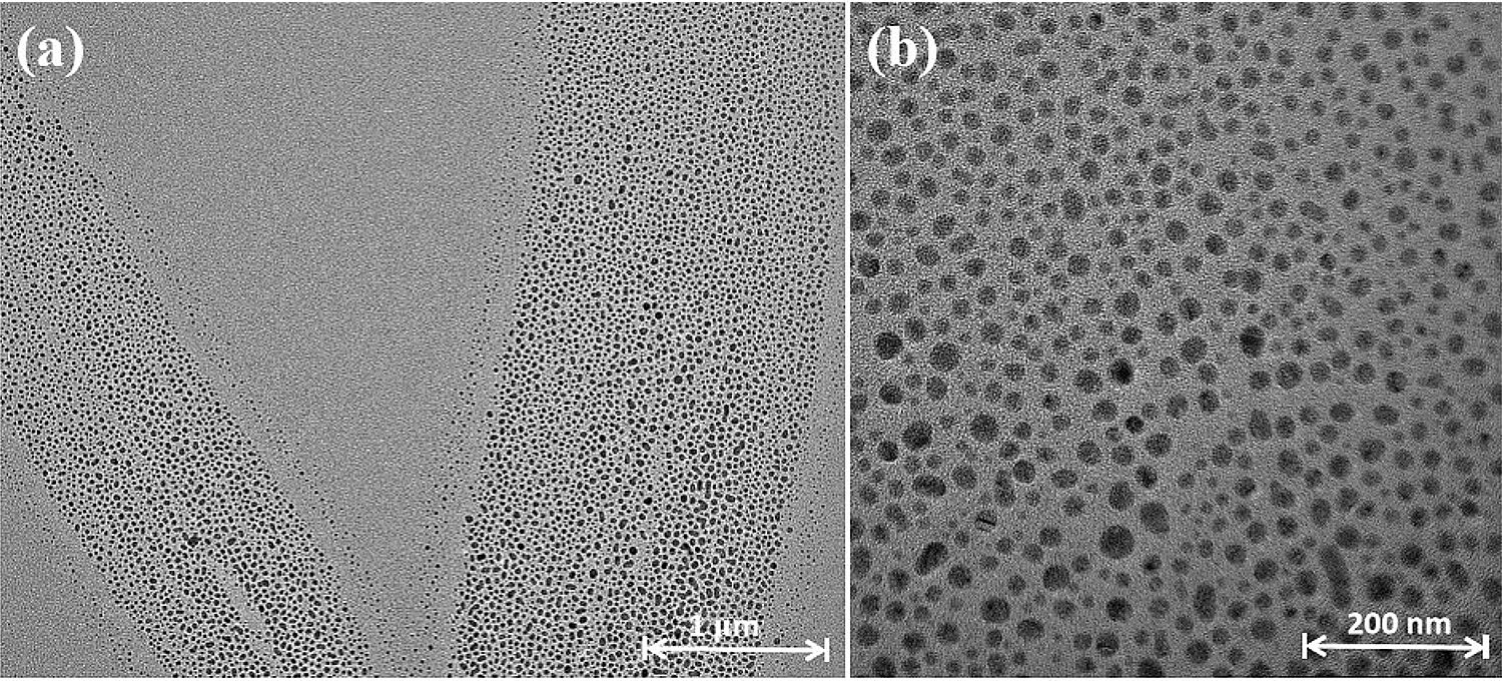
HRTEM images of Ag_2_O NPs (60 °C/10 min) with (**a**) 1 μm scale (**b**) and 200 nm scale.

Furthermore, the uniform morphology of Ag_2_O NPs (60 °C/10 min) and spherical crystal shape with quite small size were observed by HRTEM images in Fig. 3. The smaller particle size is usually more beneficial to the antibacterial reaction. Because the particle size is smaller, much more particles will be easily adsorbed on the surface of the bacterial cell membrane, and then successfully attack the cell to destroy the physiological function in the cell. It may be a key reason to explain that Ag_2_O NPs (prepared at 60 °C/10 min) have the best antibacterial performance among all as prepared Ag_2_O NPs.

The thermogravimetric analysis of Ag_2_O prepared at 60 °C/10 min is shown in Fig. S2. According to the outermost electronic arrangement of Ag, it is the most stable when the outermost electronic arrangement is 4d^10^, which is the reason why the order of stability of silver oxides is Ag_2_O > Ag_2_O_2_. At 438.85 °C, as shown in the Fig. S2, it can be demonstrated that Ag_2_O began to decompose to Ag, releasing O_2_ and reducing the sample quality. At 473.17 °C, Ag_2_O was completely decomposed. The melting point of silver is 967.18 °C, thus the sample quality is basically constant thereafter. During the temperature rise of 40–800 °C, the mass loss of Ag_2_O is only 7.847%, which evidences that the materials have excellent thermal stability.

### Antibacterial performances of Ag_2_O NPs

The antibacterial performances of Ag_2_O NPs on *E. coli* have been evaluated via monitoring the emerging and extent of inhibition zones, and measuring OD value at 600 nm during the process of their interaction.

#### Paper-disk diffusion test

As shown in Fig. S3, under the same concentration, among three different antibacterial agents, Ag_2_O NPs, ethanol and H_2_O_2,_ Ag_2_O NPs resulted in the most obvious and well-proportioned inhibition zone on the solid medium of *E. coli* (Fig. S3a). The diameter of inhibition zone were 1.91 cm (*E. coli*) and 1.96 cm (*S.* *aureus*), respectively. In contrast, no clear inhibition zone appeared when using ethanol and H_2_O_2_ soaked filter paper. Such better antibacterial action of Ag_2_O NPs than that of other two traditional disinfectants was also observed in *S.* *aureus* group (Fig. S3b). Thus, it is inferred that compared with two kinds of traditional antibacterial agents (H_2_O_2_ and ethanol), as-prepared Ag_2_O NPs have better inhibition performance to *E. coli* and *S.* *aureus*.

#### MIC and MBC test

MIC and MBC are generally used to evaluate the ability of an antibacterial agent. After testing, it could be concluded that as prepared Ag_2_O NPs gave the MIC of 30 μg mL^−1^, much lower than that of commercially available particles which gave the MIC of around 800 μg mL^−1^^29^, while the MBC was 40 μg mL^−1^ (Table S1, Figs. S4, S5), strongly evidencing the advantage of the quite low using dose.

#### Quantitative antibacterial activity test

**Figure 4:**
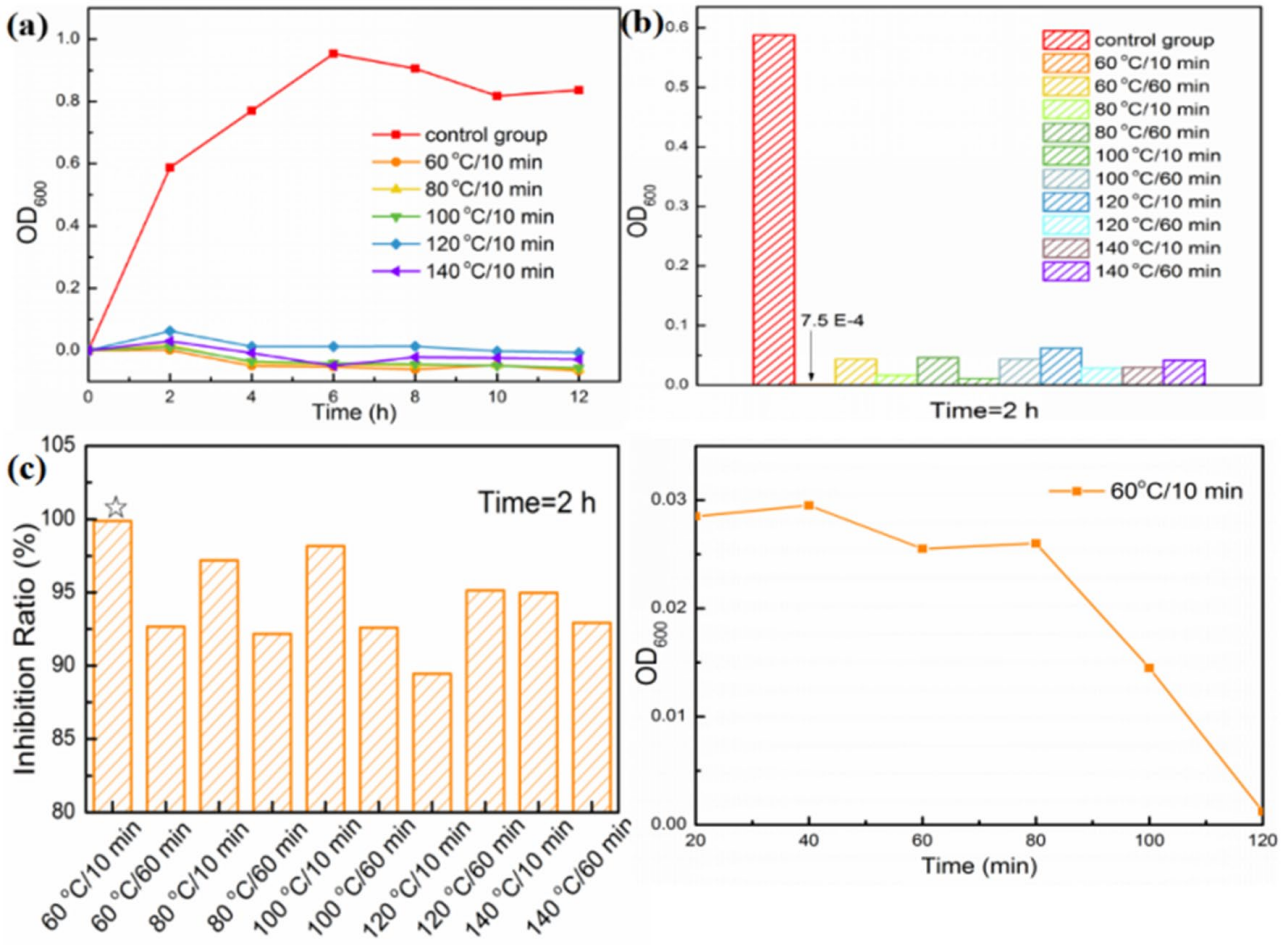
(**a**) Growth curves of *E. coli* co-cultured with Ag_2_O NPs (60–140°C/10 min) within 12 h. (**b**) OD_600_ of *E. coli* co-cultured with Ag_2_O NPs under different preparation conditions after 2 h. (**c**) Inhibition ratios of *E. coli* co-cultured with Ag_2_O NPs under different preparation conditions after 2 h. (**d**) Growth curve of *E. coli* co-cultured with Ag_2_O NPs (60 °C/10 min) within 2 h.

The inhibition growth curves were used to study the dynamics of bacterial growth, and evaluated the antibacterial properties of Ag_2_O NPs with different preparation conditions. Overall, as-prepared Ag_2_O NPs showed noteworthy antibacterial activities in Fig. 4a, which shows the growth curves of the culture medium of *E. coli* co-cultured with different Ag_2_O NPs (60 °C , 80 °C, 100 °C, 120 °C, 140 °C/10 min) at 37 °C for varied time intervals (up to 12 h). Particularly, considering the balance of efficiency and economic, the best antibacterial activity was observed at 60 °C/10 min group with almost 100% antibacterial ratio was obtained after only 2 h (Fig. 4b,c). The higher antibacterial efficiency is likely owing to the smaller particles and uniform morphology, which is consistent with the results of XRD analysis (Fig. 1), SEM images (Fig. 2) and HRTEM measurements (Fig. 3). Some quantitative results of antibacterial activity are provided in supplementary information (Figs. S6–S9).

**Figure 5:**
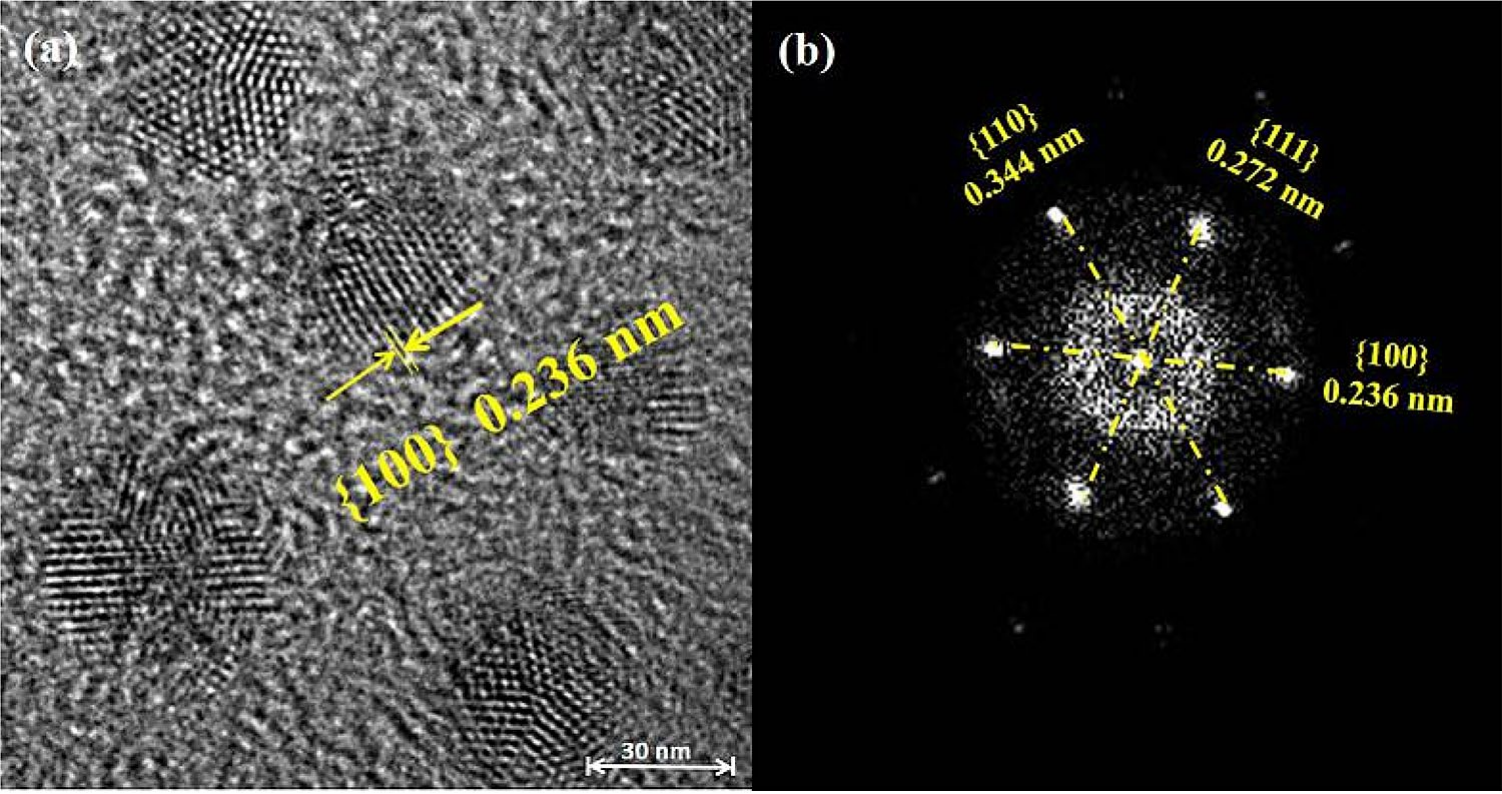
(**a**) HRTEM image of Ag_2_O NPs (60 °C/10 min), (**b**) Corresponding SEAD pattern of Ag_2_O NPs (60 °C/10 min).

**Figure 6:**
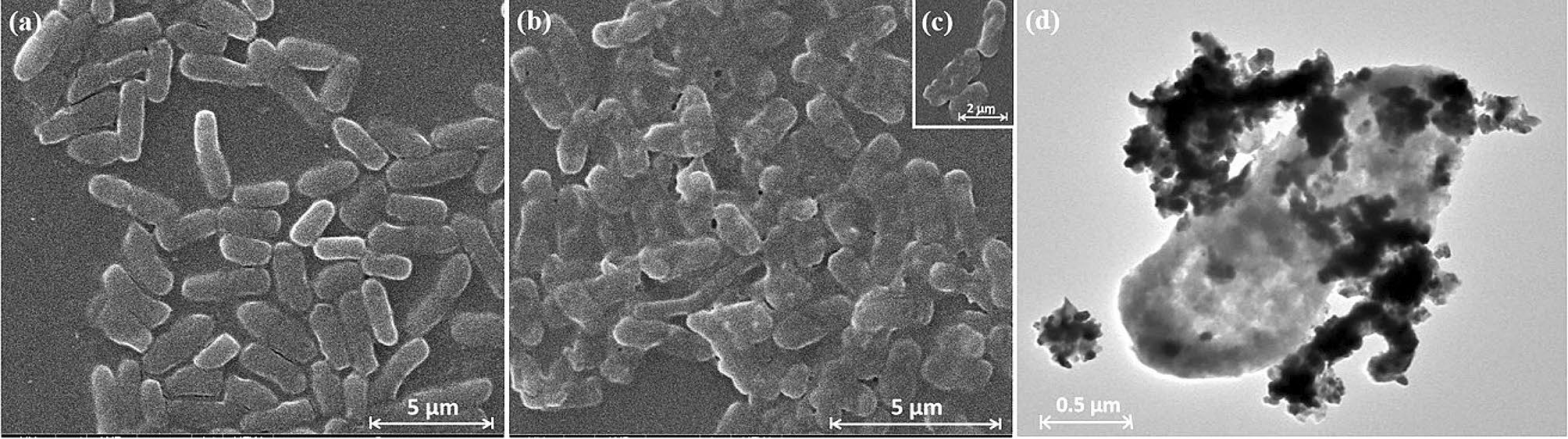
E-SEM images of *E. coli* cells (**a**) before and (**b**) after sterilization and a partial enlargement (inset) co-culturing with Ag_2_O NPs (60 °C/10 min) for 2 h. TEM image of *E. coli* cell and Ag_2_O NPs after reaction (**d**).

To understand why Ag_2_O NPs (60 °C/10 min) have higher activity, their crystalline features and interaction with cells were studied. It can be known that the {100} facet is much more exposed in Ag_2_O NPs (60 °C/10 min) than that in other Ag_2_O NPs as inferred XRD patterns (Fig. 1). As shown in Fig. 6a, Ag_2_O NPs (60 °C/10 min) have {100} facet exposure via analyzing crystal lattice, and it can be clearly seen that a single Ag_2_O NP presents a uniform spherical shape. In addition, the results of selected area electron diffraction (SAED) pattern are shown in Fig. 5b, presenting six obvious diffraction spots connected in a circle and are symmetrical, which are corresponding to the presence of {100}, {111}, {110} plane axis, as well as the XRD results. In the present work, due to the different atomic arrangement, Ag_2_O NPs have several crystal planes, which may cause differences in the release rate and concentration of Ag^+^, further influencing their antibacterial performances of antibacterial. According to the previous work, the {100} facets are more reactive^28^. Moreover, the Ag^+^ ions are more likely to adsorb on the {100} surface. Because the charged ions can be preferentially adsorbed on the cell membrane, and the high reactivity of the {100} facet makes Ag_2_O NPs more easily adsorbed on the surface of *E. coli* membrane to participate in the afterwards antibacterial reaction. From Fig. 5a, it can be concluded that higher reactive {100} facet indeed highly exposed in the 60 °C/10 min Ag_2_O NPs sample.

#### Inhibition–sterilization process

An interesting phenomenon is the OD_600_ value achieved around 0 at 2 h (Fig. 4a,b). In order to further studying the antibacterial process in the first 2 h, the growth curve of Ag_2_O NPs (60 °C/10 min) within 2 h was conducted. As shown in Fig. 4d, the concentration of bacteria maintains the same level for the first 80 min. In the next 40 min, the antibacterial agent begins to play a significant role, and the number of the bacteria cell is damaged to the initial concentration level in the end of sterilization step. The result above reveals that the Ag_2_O NPs takes effect after at least 1 h. More importantly, it can be concluded that the whole antimicrobial process may consist of two steps, the first one is inhibition procedure, which means, the reproduced capacity of bacteria was destroyed by Ag_2_O NPs, thus the number of cells no longer increases. Following this step, Ag_2_O NPs sterilized the rest level of bacteria, which is the sterilization step.

The morphological changes of *E. coli* cells can be represented via E-SEM images in Fig. 6a–c. At the original state, the cells had the full membrane with smooth surface, and showed good dispersity (Fig. 6a). However, after co-culturing with Ag_2_O NPs (60 °C/10 min) for 12 h, it can be evidently seen that the membranes of all cells became indistinct and messy, and severe adhesion occurred among them (Fig. 6b). The destruction of cell membrane belongs to the “inhibition” part, which is the initial antibacterial action of Ag_2_O NPs against *E. coli* cells. And then the intercellular dissolved matters flowed out gradually such as proteins, sugars, and so on. Compared to the average size of 2 µm of *E. coli* cells, the size of Ag_2_O NPs is quite smaller, which makes more react sites on the membrane of *E. coli*^13^. From Fig. 6d, it can be clearly seen that some of the Ag_2_O NPs attached to the surface of *E. coli* cell after the reaction, and the others entered the inside of the bacterial cell. After the reaction, the Ag_2_O NPs showed agglomeration, and the bacterial cell membrane was obviously incomplete. In a word, the antibacterial agent destroyed the membrane, which was seen as the “protective shield” of intact cells, making the bacteria lose the basic living viability, then leading to the ultimate death of the cells. This result is consistent with the two-step inhibition–sterilization antibacterial process as we proposed above.

In order to help analyze the changes of the groups of *E. coli* before and after the bactericidal reaction, the FTIR measurement of *E. coli* before and after the reaction were conducted and the results were shown in Fig. S10. The infrared spectrum showed that phospholipid bilayer, which is the important component of cell membrane, protein and DNA were destroyed to some extent, suggesting that Ag_2_O NPs attacked these parts of *E. coli*, leading to cell apoptosis in the end. However, the changes of the stretching vibration summit of the FTIR appear superposition. For example, the stretching vibration peak of MDA is around 1725 cm^−1^, which coincides with the area of the phosphate diester group, thus the changes cannot be accurately observed. Therefore, infrared spectra can only be used for reference.

Overall, the inhibition–sterilization antimicrobial process was explained from the results above, which is showed in Scheme 1b,c. It can be inferred that when Ag_2_O NPs began to contact with bacterial cells, the destruction of DNA and proteins may not effectively weaken the reproductive capacity of the bacteria. The number of damaged cells is equal to the number of reproduced cells, which is defined as the antibacterial inhibition step. However, after a period of continuous contact, the reproductive capacity of *E. coli* drops dramatically, even the newly reproduced bacterial cells begin to be destroyed, thereby the growth curve shows a sharp decline trend. This stage is sterilization step.

**Figure 7:**
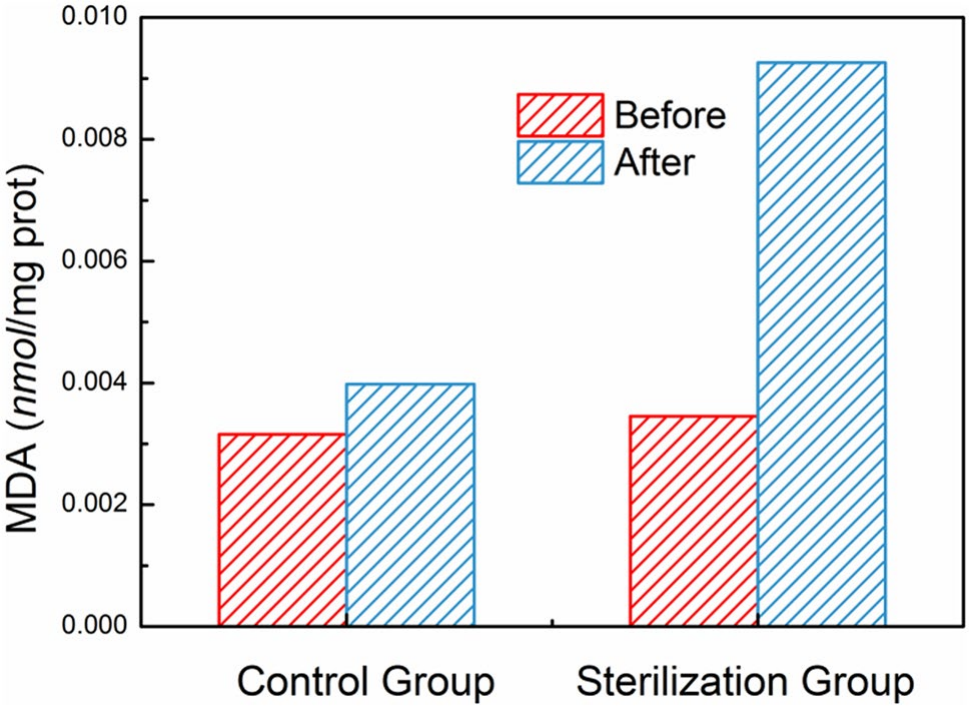
MDA concentrations before and after sterilization.

When the cell membrane subjected to oxidative stress, MDA will produce spontaneously from the cell. Therefore, MDA is regarded as a symbol of the destruction of cell membrane. In order to further proof that the Ag_2_O NPs damaged the cell membrane and then entered into the cell to interact with the intracellular substance, the MDA concentrations in the cell were evaluated^32,33^. As shown in Fig. 7, in the sterilization group, after exposing to Ag_2_O NPs (60 °C/10 min), the concentration of MDA in *E. coli* cells (0.00926 nmol/mg prot) is around 3 times higher than the initial concentration (around 0.003 nmol/mg prot), which represents the MDA concentration before adding the antibacterial agent. Therefore, it is a strong evidence to prove the destruction of cell membrane from the MDA concentration change, which is consistent with the results of E-SEM and FTIR analyses as well as the inhibition–sterilization process we proposed above.

## Conclusion

To sum up, a series of Ag_2_O NPs with small size, uniform morphology and excellent thermostability were synthesized. The synthesis reaction temperature and reaction time critical influence on the crystalline and morphological properties of Ag_2_O NPs and thus impact their antibacterial performances. A high antibacterial ratio of 100% can be reached within 40 min after an 80 min inhibition stage. More importantly, the inhibition–sterilization antibacterial process was proposed via growth curves, E-SEM measurements, FT-IR spectrum and MDA concentration detection. In the future, the study on antibacterial process by Ag_2_O NPs will be conducted by other kinds of bacteria, such as *Staphylococcus aureus, Pseudomonas aeruginosa* and so on, especially those widely presenting in environment. Also, more endeavors for the interfacial and molecular level experiments are needed to unveil the detailed inhibition–sterilization process at microcosmic level.

## Supplementary Information

Supplementary Information

## References

[1] Morens DM, Folkers GK, Fauci AS (2008). Emerging infections: A perpetual challenge. Lancet Infect. Dis..

[2] Tattevin P, Mallet MB, Gougeon AJ (2012). Emergency of resistance to antibacterial agents: The role of quaternary ammonium compounds-a critical review. Int. J. Antimicrob. Aging.

[3] Jatoi AW, Kimc IS, Ni QQ (2019). Cellulose acetate nanofibers embedded with Ag NPs anchored TiO_2_ nanoparticles for long term excellent antibacterial applications. Carbohyd. Polym..

[4] Chouirfa H, Bouloussa H, Migonney V, Falentin-Daudré C (2019). Review of titanium surface modification techniques and coatings for antibacterial applications. Acta Biomater..

[5] Murugesan P, Moses JA, Anandharamakrishnan C (2019). Photocatalytic disinfection efficiency of 2D structure graphitic carbon nitride-based nanocomposites: A review. J. Mater. Sci..

[6] Li S (2018). Antibacterial hydrogels. Adv. Sci..

[7] Mahmoud H, Mahdi JS, Iman K (2018). Inorganic and metal nanoparticles and their antimicrobial activity in food packaging applications. Crit. Rev. Microbiol..

[8] Qi K, Cheng B, Yu J, Ho W (2017). Review on the improvement of the photocatalytic and antibacterial activities of ZnO. J. Alloy. Compd..

[9] He W, Zhang Y, Li J (2016). A novel surface structure consisting of contact-active antibacterial upper-layer and antifouling sub-layer derived from Gemini quaternary ammonium salt polyurethanes. Sci. Rep..

[10] Mohameda GG, Solimanb MH (2010). Synthesis, spectroscopic and thermal characterization of sulpiride complexes of iron, manganese, copper, cobalt, nickel, and zinc salts. Antibacterial and antifungal activity. Spectrochim. Acta A.

[11] Hameed AS (2016). In vitro antibacterial activity of ZnO and Nd doped ZnO nanoparticles against ESBL producing *Escherichia**coli* and *Klebsiella**pneumoniae*. Sci. Rep..

[12] Kasinathan K, Kennedy J, Elayaperumal M, Henini M, Malik M (2016). Photodegradation of organic pollutants RhB dye using UV simulated sunlight on ceria based TiO_2_ nanomaterials for antibacterial applications. Sci. Rep..

[13] Ma S, Zhan S, Jia Y, Zhou Q (2015). Superior antibacterial activity of Fe_3_O_4_–TiO_2_ nanosheets under solar light. ACS Appl. Mater. Inter..

[14] Zhu Q (2017). A novel P/Ag/Ag_2_O/Ag_3_PO_4_/TiO_2_ composite film for water purification and antibacterial application under solar light irradiation. Sci. Total Environ..

[15] Grandcolas M, Ye J, Hanagata N (2011). Combination of photocatalytic and antibacterial effects of silver oxide loaded on titania nanotubes. Mater. Lett..

[16] Sun D (2017). Transcriptome analysis reveals silver nanoparticle-decorated quercetin antibacterial molecular mechanism. ACS Appl. Mater. Inter..

[17] Rajabi A (2018). Development and antibacterial application of nanocomposites: Effects of molar ratio on Ag_2_O–CuO nanocomposite synthesised via the microwave-assisted route. Ceram. Int..

[18] Tripathi S, Mehrotra GK, Dutta PK (2011). Chitosan-silver oxide nanocomposite film: Preparation and antimicrobial activity. B. Mater. Sci..

[19] Hu Z, Chan WL, Szeto YS (2008). Nanocomposite of chitosan and silver oxide and its antibacterial property. J. Appl. Polym. Sci..

[20] Trang VT (2017). Functional iron oxide-silver hetero-nanocomposites: Controlled synthesis and antibacterial activity. J. Electron. mater..

[21] Ma J, Zhang J, Xiong Z, Yong Y, Zhao XS (2011). Preparation, characterization and antibacterial properties of silver-modified graphene oxide. J. Mater. Chem..

[22] Chu Z, Zhao T, Li L, Fan J, Qin Y (2017). Characterization of antimicrobial poly (lactic acid)/nano-composite films with silver and zinc oxide nanoparticles. Materials.

[23] Durán N, Durán M, Seabra AB, Fávaro WJ, Nakazato G (2016). Silver nanoparticles: A new view on mechanistic aspects on antimicrobial activity. Nanomed. Nanotechnol..

[24] Li P (2019). Metal-organic frameworks with photocatalytic bactericidal activity for integrated air cleaning. Nat. Commun..

[25] Allahverdiyev AM, Abamor ES, Bagirova M, Rafailovich M (2011). Antimicrobial effects of TiO_2_ and Ag_2_O nanoparticles against drug-resistant bacteria and *leishmania* parasites. Future Microbiol..

[26] Duffy LL, Osmond-McLeod MJ, Judy J, King T (2018). Investigation into the antibacterial activity of silver, zinc oxide and copper oxide nanoparticles against poultry-relevant isolates of Salmonella and Campylobacter. Food Control.

[27] Kim T (2016). Composite porous silicon-silver nanoparticles as theranostic antibacterial agents. ACS Appl. Mater. Inter..

[28] Wang X (2010). Shape-dependent antibacterial activities of Ag_2_O polyhedral particles. Langmuir.

[29] Kawashita M, Toda S, Kim H, Kokubo T, Masuda N (2003). Preparation of antibacterial silve-doped silica glass microspheres. J. Biomed. Mater. Res. A..

[30] Pizzimenti S (2013). Interaction of aldehydes derived from lipid peroxidation and membrane proteins. Front. Physiol..

[31] Whelan DR (2011). Monitoring the reversible B to A-like transition of DNA in eukaryotic cells using Fourier transform infrared spectroscopy. Nucleic Acids Res..

[32] Joseph S, Kamath PV (2008). Electrochemical deposition of Cu_2_O on stainless steel substrates: Promotion and suppression of oriented crystallization. Solid State Sci..

[33] Wang Z (2019). Dimethyl phthalate damaged the cell membrane of *Escherichia**coli* K12. Ecotox. Environ. Safe..

