# Supplementary material for: The interaction of Ag2O nanoparticles with Escherichia coli: inhibition–sterilization process
Source: Sci Rep. 2021 Jan 18;11:1703. doi: 10.1038/s41598-021-81305-5 (PMC7813836; doi:10.1038/s41598-021-81305-5)
Supplement: Supplementary file 1 — Supplementary Information [file 41598_2021_81305_MOESM1_ESM.doc]

# Supplementary Information

**The Interaction of Ag_2_O Nanoparticles with Escherichia coli: Inhibition–Sterilization Process**

*Danqing Li^a,b^, Shuai Chen^*b,c^, Ke Zhang^a^, Nan Gao^c^, Miao Zhang^a^, Gadah Albasher^d^, Jiangfan Shi^b^, Chuanyi Wang^*a^*

*^a^ School of Environmental Science and Engineering, Shaanxi University of Science and Technology, Xi’an 710021, China*

*^b^ Nano Institute of Utah and Department of Materials Science and Engineering, University of Utah, Salt Lake City 84112, Utah, USA*

*^c^ School of Pharmacy, Jiangxi Science & Technology Normal University, Nanchang 330013, Jiangxi, China*

*^d^ Department of Zoology, Science College, King Saud University, Riyadh, 11451, Saudi Arabia*

*^*^Corresponding author*

Phone: +86 13299131206 (C. Wang)

Emails: [shuai.chen@utah.edu](mailto:shuai.chen@utah.edu) (S. Chen); [wangchuanyi@sust.edu.cn](mailto:wangchuanyi@sust.edu.cn) (C. Wang)


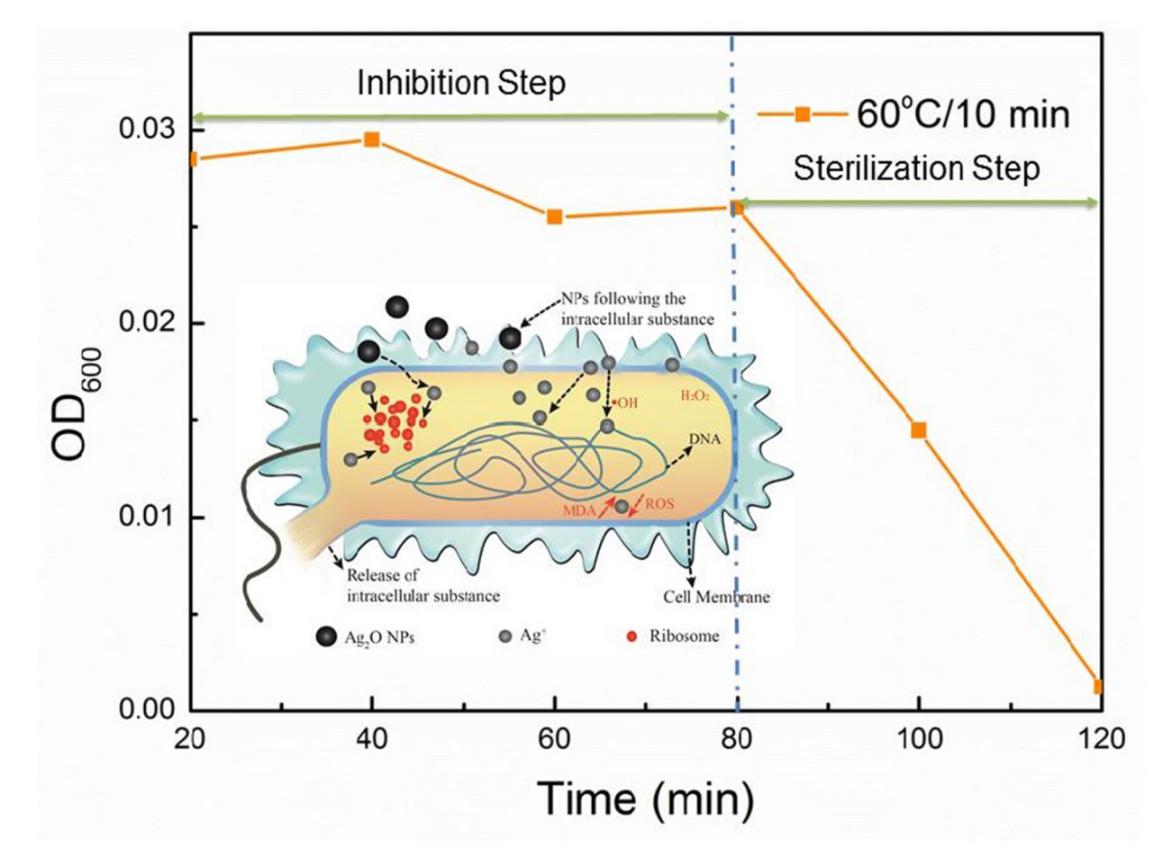
**Abstract Graphic**


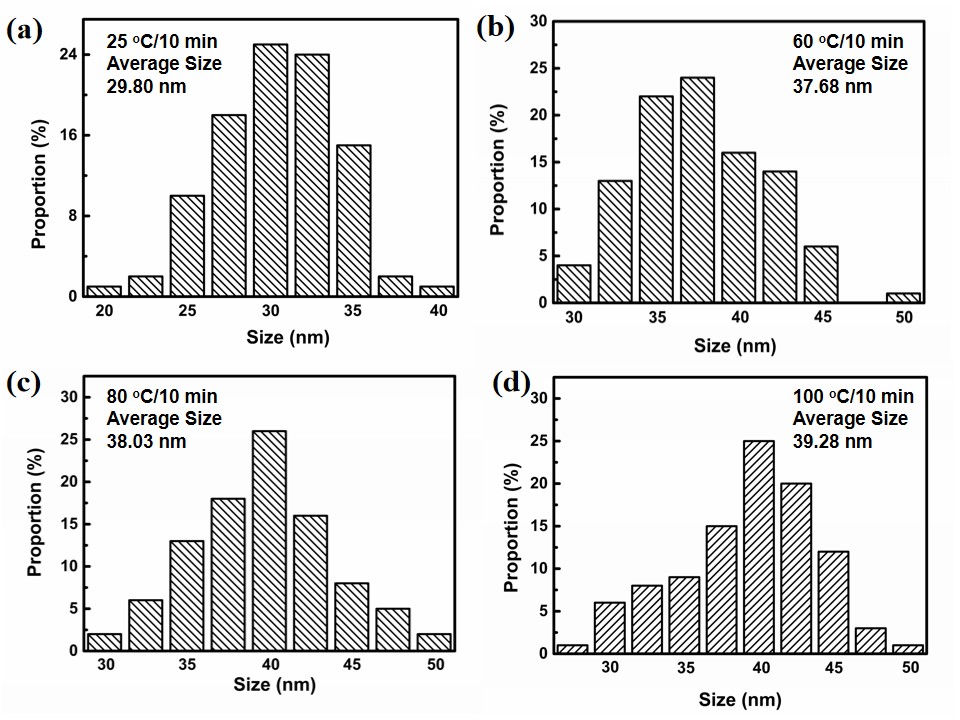


Figure S1. The size distribution of Ag_2_O NPs under different synthesis conditions of (a) 25 ^o^C/10 min, (b) 60 ^o^C/10 min, (c) 80 ^o^C/10 min, (d) 100 ^o^C/10 min.





Figure S2. Thermogravimetric (TG) analysis of Ag_2_O NPs (60 ^o^C/10 min).

**
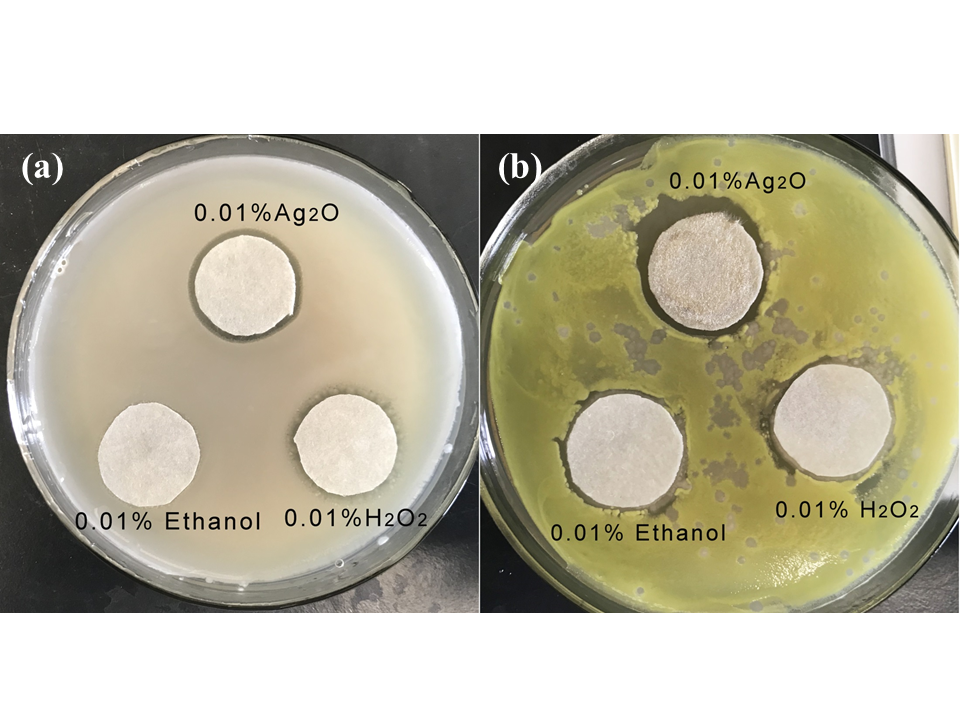
**Figure S3. Images of inhibition zone of (a) E. *coli* and (b) S. *aureus* under sterilizing agents treatment, 0.01 wt.% Ag_2_O NPs (60 ^o^C/10 min) suspension, 0.01 vol.% absolute ethanol and 0.01 vol.% H_2_O_2_, respectively.

Table S1. Values of MIC and MBC of Ag_2_O NPs (60 ^o^C/10 min) against E. *coli*.

| Ag_2_O/E ∙*coli* | MIC | MBC |
| --- | --- | --- |
| μg mL^-1^ | 30 μg mL^-1^ | 40 μg mL^-1^ |


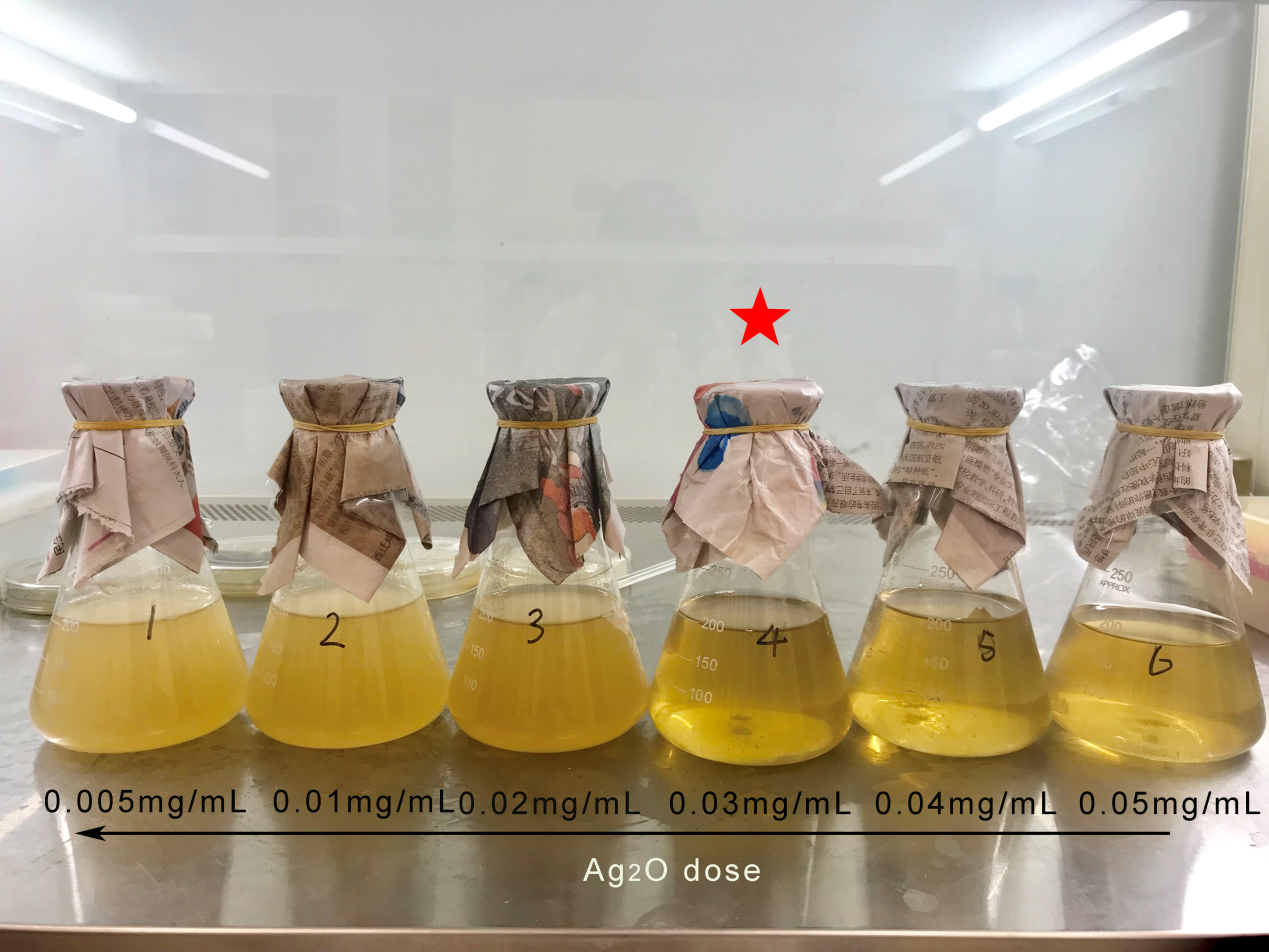
Figure S4. The image of MIC measurement result of Ag_2_O NPs (60 ^o^C/10 min).


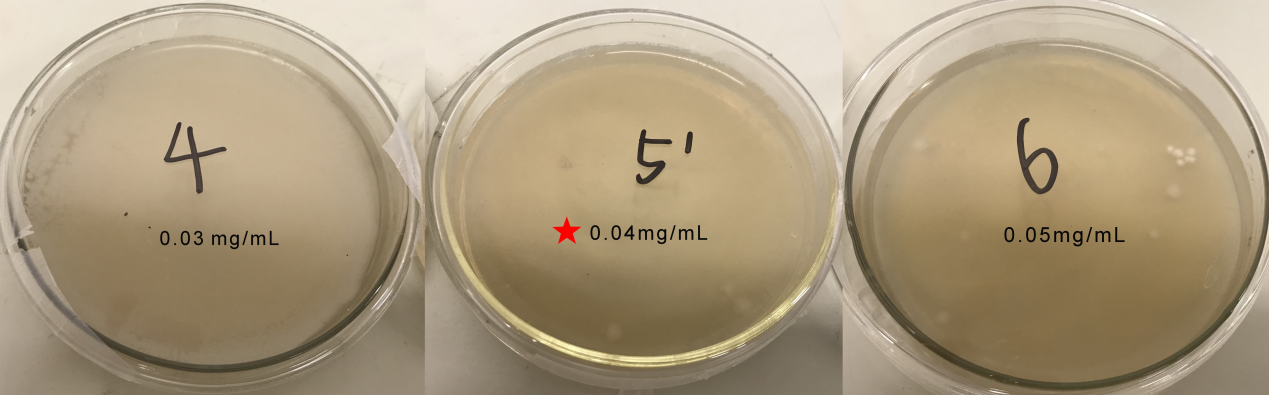
Figure S5. The images of MBC measurement result of Ag_2_O NPs (60 ^o^C/10 min).





Figure S6. Growth curves of E.*coli* co-cultured with Ag_2_O NPs (80 ^o^C/10-90 min) for 12 h.





Figure S7. Growth curves of E.*coli* co-cultured with Ag_2_O NPs (80 ^o^C/ 10 min, 80 ^o^C/ 60 min) for 12 h.





Figure S8. OD_600_ of E. *coli* co-cultured with Ag_2_O NPs (80 ^o^C/ 10 min-90 min) after 12 h.


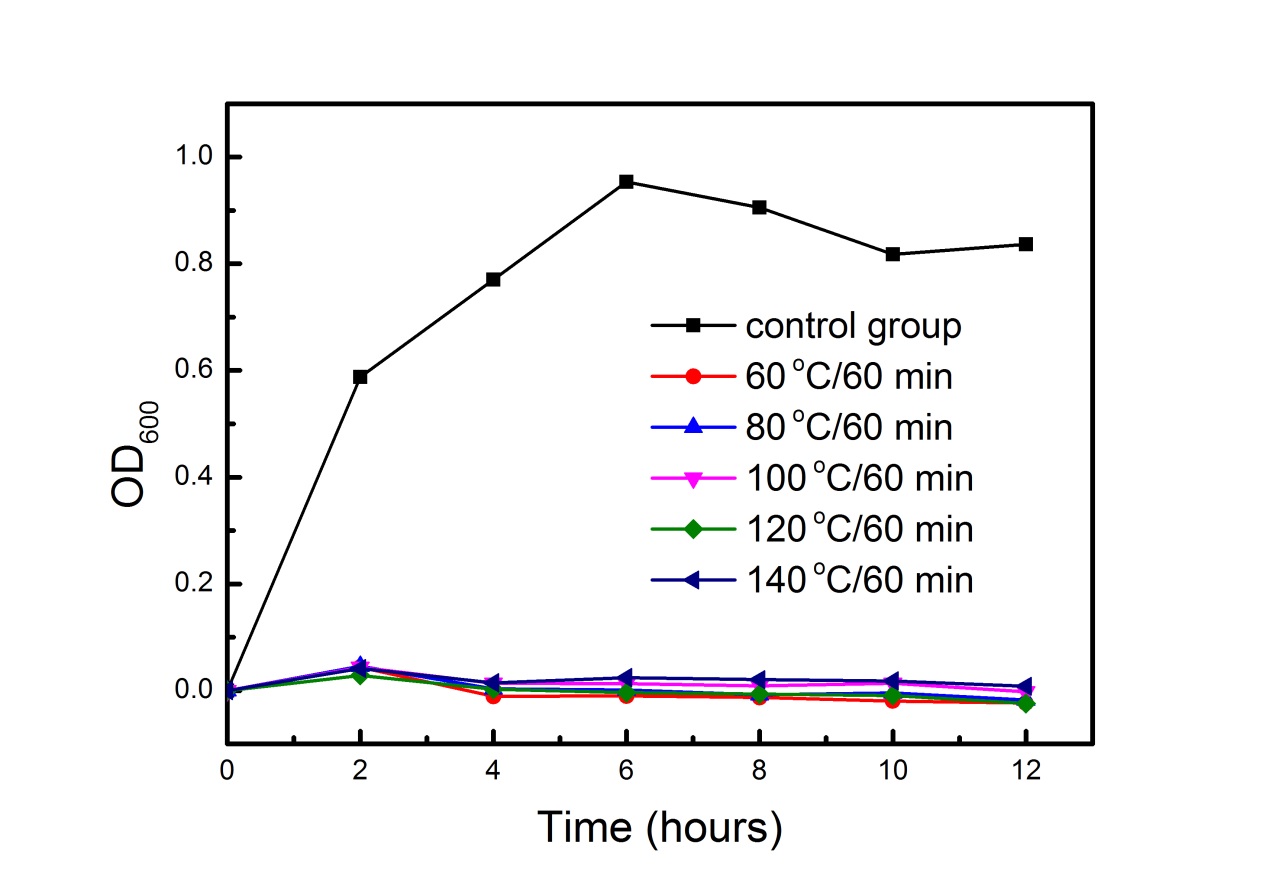


Figure S9. Growth curves of E.*coli* co-cultured with Ag_2_O NPs (60 ^o^C-140 ^o^C / 60 min) for 12 h.


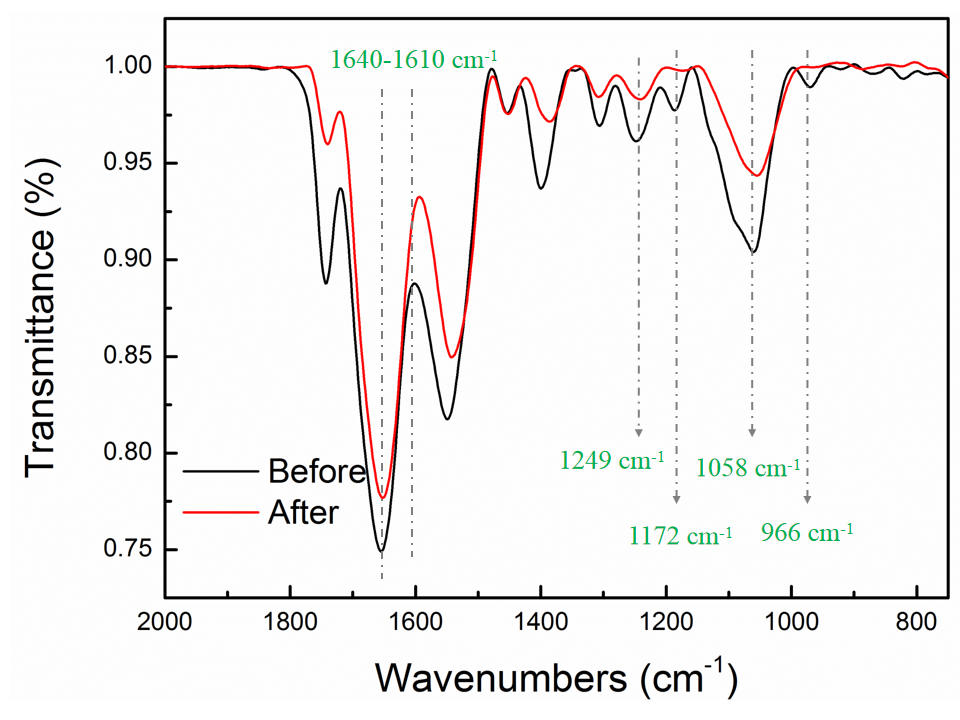
Figure S10. FTIR spectrums of E. *coli* before and after sterilization.

As seen in Figure S10, after the action of sterilizing agent, the peak intensity of the group of E. *coli* was significantly reduced. The peaks located at 1172 cm^-1^, 1058 cm^-1^ and 966 cm^-1^ decrease obviously, which represents the anti-symmetric vibrational peak of phosphodiester group and the phosphate bond stretching vibration peak, respectively.^28^ The three peaks present important components of the cell membrane. This result confirmed the destruction of the cell membrane further and supported the inhibition process acted firstly. In addition, it can be seen that the spectrum shows slightly decrease from 1640 to 1610 cm^-1^, which is the characteristic absorption of the b-sheet structure in the amide I region. The b-sheet content in the protein secondary structure reduced from the Figure S10.^29, 30^ It is a direct proof to indicates that protein is destoryed and the structure is incomplete. It is well known that protein is the basic organisms that make up cell, which is a significant barrier to life activities. At last the serious destruction of protein may lead to the cell death. More importantly, nucleic acids are responsible for the storage, replication and transmission of genetic information, as well as DNA, which plays an important role in the process of protein synthesis. The spectral region of 1300–1180 cm^-1^ contains the characteristic absorptions of A-form and B-form DNAs.^31^ It can be clearly seen the change during this region is remarkable, which means Ag_2_O NPs destroyed the DNA in E.*coli.* It is a direct evidence of antibacterial mechanism study.
